# The Cervical Edge

**Published:** 1885-11

**Authors:** J. Smith Dodge


### THE CERVICAL EDGE.

BY J. SMITH DODGE, JR., M. D., D. D. S.

May it not be that the mysterious tendency to decay at the cervical border of fillings is largely due to defective trimming? Since I formed the habit of testing every proximal filling by passing floss silk between the gum and the neck, and drawing it along the tooth towards the free edge, I have been continually surprised to find how difficult it is to obtain perfect smoothness at the cervical border. Now any abrupt projection of the filling at this part will promote decay in two ways. It will make impossible the perfect removal of fibrous food, and it will produce that acid secretion from the mucous surface which has been shown to follow prolonged irritation of the gums.

The conjecture that this is a frequent cause of cervical decay is strengthened by considering the difference of filling materials in this respect. Did anybody ever see recurrent cervical decay beside a gutta-percha filling? Certainly, very seldom; while fillings of oxy-chloride or phosphate permit it readily. Now gutta-percha is so easily trimmed with a warm spatula that it is almost certainly well finished at this point. And if it is not, gutta-percha has no irritating effect on the gum, and is so flexible that it will much less firmly retain foreign particles, in both which respects the mineral fillings named are greatly different. Then again, consider the well-recognized contrast between fillings of soft foil and those of cohesive gold. It was very rare that one found cervical decay by a well-made soft foil filling, while it has from the first been the besetting sin of cohesive gold. May it not be because the projecting mass of soft foil yields so readily to the customary stroke of the flat burnisher between the teeth, that no abrupt projection is left? It is certain that since the matter took this shape in my mind (several years ago), my gold fillings have been almost exempt from cervical failure.

But perhaps the strongest confirmation is found in teeth filled with amalgam. How many teeth have I seen, in the mouth or out, with large amalgam fillings sharply projecting where the cervical wall had been, but flanked on that side by a great and fatal chasm. Of course, such an edge of amalgam, once well hardened, presents exactly the conditions for retaining food and for constantly irritating the gum. Any dentist who investigates this for the first time will be surprised to find how naturally, almost inevitably, a filling finished with spatulas projects at the cervical margin. But since I adopted the use of silk for trimming next the gum, my amalgam fillings have in hardly an instance permitted secondary cervical decay.

My object is not to present this as the complete explanation of cervical failure, but to suggest that this may be the reason why many fillings, otherwise thoroughly good, prove insufficient at this critical point.

